# Randomised clinical trial: a 1-week, double-blind, placebo-controlled study of pancreatin 25 000 Ph. Eur. minimicrospheres (Creon 25000 MMS) for pancreatic exocrine insufficiency after pancreatic surgery, with a 1-year open-label extension

**DOI:** 10.1111/apt.12236

**Published:** 2013-02-05

**Authors:** C M Seiler, J Izbicki, L Varga-Szabó, L Czakó, J Fiók, C Sperti, M M Lerch, R Pezzilli, G Vasileva, Á Pap, M Varga, H Friess

**Affiliations:** * Department of General, Visceral and Vascular Surgery, Josephs-Hospital WarendorfWarendorf, Germany; † Department of General, Visceral and Thoracic Surgery, University Hospital Hamburg-EppendorfHamburg, Germany; ‡ Department of Gastroenterology, Szent Pantaleon HospitalDunaújváros, Hungary; § First Department of Medicine, University of SzegedSzeged, Hungary; ¶ Sopron Medical SMO Erzsébet HospitalSopron, Hungary; ** Department of Surgery, Oncology, and Gastroenterology, University of PaduaPadua, Italy; †† Department of Medicine A, Medical University of Greifswald Ernst-Moritz-ArndtGreifswald, Germany; ‡‡ Department of Digestive Diseases and Internal Medicine, Sant'Orsola-Malpighi HospitalBologna, Italy; §§ Department of Gastroenterology and Hepatology, MHAT Rousse ADRousse, Bulgaria; ¶¶ Department of Gastroenterology, Outpatient Clinic, Europ-Med CoBudaörs, Hungary; *** 3rd Department of Medicine and Gastroenterology, Réthy Pál HospitalBékéscsaba, Hungary; ††† Department of Surgery, Klinikum rechts der Isar, Technical University MunichMunich, Germany

## Abstract

**Background:**

Pancreatic exocrine insufficiency (PEI) often occurs following pancreatic surgery.

**Aim:**

To demonstrate the superior efficacy of pancreatin 25 000 minimicrospheres (Creon 25000 MMS; 9–15 capsules/day) over placebo in treating PEI after pancreatic resection.

**Methods:**

A 1-week, double-blind, randomised, placebo-controlled, parallel-group, multicentre study with a 1-year, open-label extension (OLE). Subjects ≥18 years old with PEI after pancreatic resection, defined as baseline coefficient of fat absorption (CFA) <80%, were randomised to oral pancreatin or placebo (9–15 capsules/day: 3 with main meals, 2 with snacks). In the OLE, all subjects received pancreatin. The primary efficacy measure was least squares mean CFA change from baseline to end of double-blind treatment (ancova).

**Results:**

All 58 subjects randomised (32 pancreatin, 26 placebo) completed double-blind treatment and entered the OLE; 51 completed the OLE. The least squares mean CFA change in the double-blind phase was significantly greater with pancreatin vs. placebo: 21.4% (95% CI: 13.7, 29.2) vs. −4.2% (−12.8, 4.5); difference 25.6% (13.9, 37.3), *P* < 0.001. The mean ± s.d. CFA increased from 53.6 ± 20.6% at baseline to 78.4 ± 20.7% at OLE end (*P* < 0.001). Treatment-emergent adverse events occurred in 37.5% subjects on pancreatin and 26.9% on placebo during double-blind treatment, with flatulence being the most common (pancreatin 12.5%, placebo 7.7%). Only two subjects discontinued due to treatment-emergent adverse events, both during the OLE.

**Conclusions:**

This study demonstrates superior efficacy of pancreatin 25 000 over placebo in patients with PEI after pancreatic surgery, measured by change in CFA. Pancreatin was generally well tolerated at the high dose administered (EudraCT registration number: 2005-004854-29).

## Introduction

Partial or total pancreatic resection may be used in the management of pancreatic cancer or chronic pancreatitis. Radical pancreatic surgery is recommended as the first stage of treatment for early, resectable pancreatic cancer, and palliative resection may be carried out to relieve symptoms in later-stage disease.^1^, ^2^ The type of surgery is dependent on tumour location; common procedures include pylorus-preserving pancreaticoduodenectomy, Whipple procedure (pancreaticoduodenectomy with antrectomy) and distal pancreatectomy, while total pancreatectomy is less frequently performed.^1^ Pancreatic resection may be required in some cases of chronic pancreatitis for the relief of pain that is refractory to conservative interventions. In addition to the procedures described above, pancreaticojejunostomy and/or duodenum-preserving pancreatic head resection may be utilised in chronic pancreatitis.^3^, ^4^

In most of the procedures described above, some pancreatic tissue is preserved, and there is likely to be some residual exocrine function. However, pancreatic resection results in pancreatic exocrine insufficiency (PEI) in many patients, and those undergoing total pancreatectomy are devoid of any pancreatic function.^5^–^8^ PEI leads to the maldigestion of food and the malabsorption of nutrients, and the symptoms include steatorrhoea, abdominal pain, flatulence, weight loss and malnutrition.^9^ The severity of PEI is variable and determined by factors such as the extent of resection, the functional capacity of the remaining pancreatic tissue, the influence of the underlying disease and changes in anatomy resulting in alterations of normal intestinal physiology, transit and motility.^5^, ^8^ The standard of care for PEI, regardless of its aetiology, is pancreatic enzyme replacement therapy.

Clinical trials have demonstrated the efficacy and safety of different formulations of pancreatin (pancrelipase) enteric-coated minimicrospheres (Creon MMS; Abbott Products GmbH, Hannover, Germany) for the treatment of PEI due to a number of different disorders including cystic fibrosis, chronic pancreatitis and pancreatic surgery.^10^–^15^ The objective of this double-blind, randomised, placebo-controlled study was to demonstrate the superior efficacy of 9–15 capsules per day of pancreatin 25 000 minimicrospheres over placebo in treating PEI after pancreatic surgery. This formulation and dose have not been tested in clinical studies previously in this patient population. The efficacy and safety of long-term pancreatin treatment were also assessed in a 1-year, open-label extension (OLE) period.

## Materials And Methods

This was a 1-week, double-blind, randomised, placebo-controlled, parallel-group study with a long-term OLE. The study was registered at EudraCT: number 2005-004854-29. The study was carried out at centres in Bulgaria (*n* = 2), Germany (*n* = 4), Hungary (*n* = 8) and Italy (*n* = 3) between April 2008 and July 2011, and 15 of these 17 centres contributed patients. It was conducted in accordance with the EU Clinical Trial Directive 2001/20/EC, the International Conference on Harmonization guideline for Good Clinical Practice 1996 and the principles of the Declaration of Helsinki. The study protocol and informed consent form were approved by Ethics Committees complying with local regulatory requirements. All subjects provided voluntary written informed consent before any study-related procedures were carried out.

### Participants

Participants were enrolled by study investigators. Men and women ≥18 years old were eligible if they had severe PEI due to partial or total pancreatic resection performed at least 6 months prior to study start. Severe PEI was defined as a baseline coefficient of fat absorption (CFA) <80%. Subjects were required to be in a stable condition after pancreatic surgery (Karnofsky index ≥70), and those having surgery for cancer were also required to have finished adjuvant chemotherapy. Women were required to be non-lactating, and if of child-bearing potential, had to agree to use an effective method of contraception that had been used for at least 3 months prior to study start. Exclusion criteria were: recurrent tumour; surgery due to acute necrotising pancreatitis; ileus or acute abdomen; current excessive intake of alcohol or drug abuse; hypersensitivity to porcine proteins/pancreatin; investigational drug intake within 30 days prior to study entry; pregnancy.

Use of other pancreatic enzyme preparations during the study was prohibited. Concomitant medications influencing duodenal pH, gastric emptying and bile secretion were permitted if the dose was stable.

### Study design

**Figure 1:**
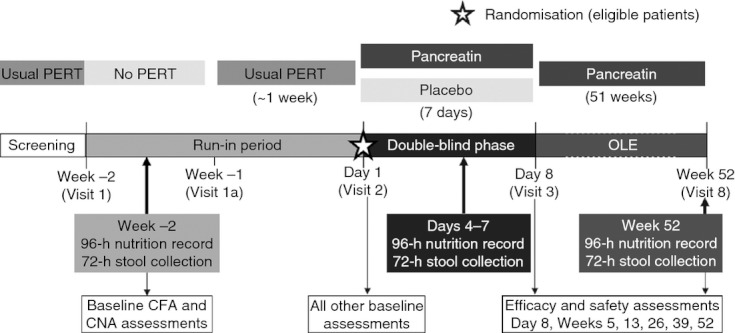
Study design. CFA, coefficient of fat absorption; CNA, coefficient of nitrogen absorption; OLE, open-label extension; PERT, pancreatic enzyme replacement therapy.

The study comprised a screening visit followed by a 2-week run-in period, a 1-week double-blind phase, and a 51-week OLE (Figure 1). To measure stool fat and stool nitrogen, 72-h stool collections were carried out during the run-in period, the double-blind phase (Days 5–7) and the last week of the OLE period (or last week before a planned drop-out). To allow assessment of fat and nitrogen intake, subjects recorded their entire nutritional intake for 96 consecutive hours, starting 24 h before each stool collection period. A dietician provided detailed instructions on how to collect stools and complete the nutrition record, and advised subjects on how to ingest 80–100 g dietary fat per day during stool collection. Patients were instructed to stop taking their usual pancreatic enzyme replacement therapy before the start of the run-in period (Visit 1); they were permitted to take their normal dose of pancreatic enzymes following completion of the first stool collection up to randomisation (Day 1/Visit 2).

Stool samples and nutrition records were collected at the end of the stool collection period during the run-in period, at the end of the double-blind phase and at the end of the OLE. Nutrition records were checked for completeness and plausibility by the dietician. Missing entries were completed with the help of the subject, if possible. Stool samples were analysed by central laboratories (Eurofins, France and ABL, Assen, The Netherlands), and stool fat was assessed according to the method of van de Kamer.^16^

### Treatment

For the double-blind phase, patients were randomised 1:1 to oral pancreatin (Creon 25000 MMS) or placebo and received 9–15 capsules per day (3 with each of 3 main meals, 2 with each of 2–3 snacks). The pancreatin dose was therefore 75 000 Ph. Eur. lipase units per main meal and 50 000 per snack. Patients were allocated to treatment by a randomisation list generated by Abbott using a block size of 10. Pancreatin and placebo capsules were identical to maintain blinding. Patients, investigators and all other personnel involved in the conduct of the study were blind to treatment. Investigators were blind to the results of the stool fat analyses except for the one at screening (this result was required by the investigator to determine whether or not the subject met the key inclusion criterion of CFA <80%).

Participants were instructed to start taking study medication with their first meal on Day 1 of the double-blind period, after the clinic visit. In the OLE, all subjects received pancreatin at the above dose. The actual number of capsules taken by subjects was not recorded.

### Efficacy assessments

The primary efficacy measure was change in CFA from baseline to end of double-blind treatment. The CFA was assessed during run-in (to determine baseline value) and at the end of the double-blind and OLE phases, and was calculated using the following equation: CFA (%) = 100 [(mean fat intake − mean stool fat)/mean fat intake]. Daily dietary fat intake was calculated by the dietician from the net weight of fat in the nutrition record using suitable software.

The coefficient of nitrogen absorption (CNA) was calculated during run-in (baseline) and at the end of the double-blind and OLE phases as follows: CNA (%) = 100 [(mean nitrogen intake − mean stool nitrogen)/mean nitrogen intake]. Daily dietary nitrogen intake was calculated by the dietician from the net weight of dietary protein intake in the nutrition record using suitable software. Stool fat, stool nitrogen and stool weight were also assessed during run-in (baseline) and at the end of the double-blind and OLE phases.

The following clinical symptoms were assessed at all visits (baseline, end of double-blind phase, Weeks 5, 13, 26, 39 and end of OLE): stool frequency (average number per day); average stool consistency (hard, formed/normal, soft or watery); flatulence (none, mild, moderate or severe); and abdominal pain (none, mild, moderate or severe).

Body weight and body mass index (BMI) were measured at all visits. Laboratory nutritional parameters were determined from blood samples at all visits from baseline onwards: triglycerides, total cholesterol, low-density lipoprotein cholesterol, high-density lipoprotein cholesterol, retinol binding protein, transferrin, total protein, prealbumin, albumin and vitamin E. Blood samples were analysed at Quintiles Laboratories Ltd.

The Clinical Global Impression of disease symptoms was assessed at all visits from baseline onwards by investigators asking participants to rate disease symptoms on the following scale: not present (none), present but not bothersome (mild), bothersome (moderate), interfered with normal activities (severe) or prevented subject from continuing with normal activities (incapacitating).

Quality of life was assessed using the Short Form-36 Health Survey (Medical Outcomes Trust)^17^, ^18^ at baseline (Day 1), end of the double-blind period and end of OLE (or at discontinuation). Eight component scores (physical functioning, physical role functioning, bodily pain, general health, vitality, social functioning, emotional role functioning, mental health), and two component summary scores (physical and mental) were assessed, with higher values indicating a better health state. The health transition item was also assessed, comprising five response categories including ‘about the same as 1 year ago’ and ‘somewhat better now than 1 year ago’, with smaller values indicating better health.

### Safety

Safety assessments included treatment-emergent adverse events recorded by investigators using standard medical terminology and then coded according to the Medical Dictionary for Regulatory Activities version 14.0. The severity, likely relationship with study drug (unrelated, unlikely, possible or probable), changes in study medication, outcome and whether serious or not, were also recorded.

Other safety assessments included physical examination (all visits), vital signs (all visits) and laboratory safety tests (baseline, Week 26 and Week 52): haematology (haemoglobin, haematocrit, red blood cell count, white blood cell count, platelet count, differential blood count if indicated); and biochemistry (fasting glucose, creatinine, alkaline phosphatase, total bilirubin, conjugated bilirubin, alanine aminotransferase, aspartate aminotransferase, gamma-glutamyl transferase, uric acid, calcium, phosphate, potassium, serum pancreatic lipase).

### Statistics

The statistical analysis was conducted using sas, Release 9.1 (SAS Inc., Cary, NC, USA). The sample size estimate was based on the results of a previous, unpublished study of pancreatin in pancreatectomised patients, assuming a difference of 20% [residual standard deviation (s.d.) 18%] between pancreatin and placebo for the primary efficacy variable when analysed with the analysis of covariance (ancova) model described below. With normally distributed data and a two-sided α = 0.05, 19 subjects per group were needed to achieve a statistical power of 90% for the primary outcome measure. An additional factor taken into account for the sample size calculation was to have ≥80% probability of detecting an adverse event with an incidence of 5% over 1 year. This would require 32 subjects to remain in the study after 1 year; therefore, assuming a dropout rate of 40% over 1 year, approximately 54 subjects (27 per group) were to be randomised.

The safety sample was defined as all subjects who provided consent, were randomised, and had at least one dose of pancreatin or placebo. The primary efficacy analysis was performed on the full analysis sample: all subjects in the safety sample who had at least one post-baseline efficacy measurement. The per-protocol sample comprised all patients in the full analysis sample without any major protocol deviations. The OLE full analysis sample comprised all patients receiving at least one dose of pancreatin in the OLE.

Descriptive statistics are reported for baseline demographic characteristics, efficacy outcome measures and safety assessments. For the primary outcome measure of mean change in CFA from baseline to end of the double-blind phase, the least squares mean and 95% confidence intervals (CIs) were compared between treatment groups using ancova, with the baseline value as a covariate and treatment as a factor. Significance was estimated using the *F*-test at the 5% level. ancova was also performed as an exploratory analysis for the CNA and stool fat. The statistical significance of changes in all efficacy outcomes from baseline to end of OLE was assessed using the Wilcoxon Signed Rank Test (exploratory analysis). For this analysis, the treatment received by subjects in the short double-blind phase was considered to have a negligible effect on their status after 1 year.

## Results

### Patient characteristics

**Figure 2:**
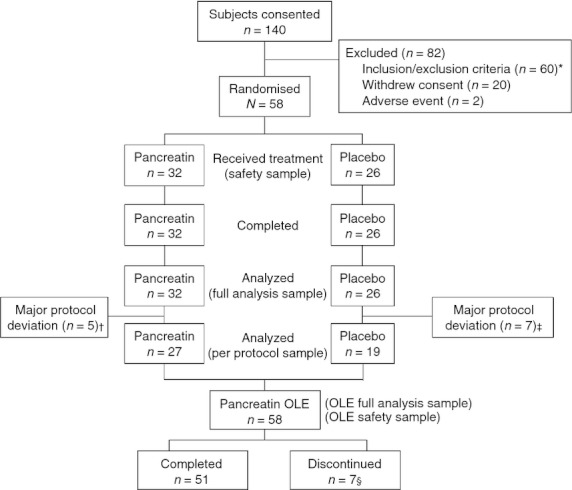
Patient disposition. OLE, open-label extension. *Majority did not meet the interim inclusion criterion of CFA <80%. †Insufficient exposure to study medication during double-blind phase (*n* = 3); insufficient treatment compliance (*n* = 1); deviation from inclusion criterion of CFA <80% (*n* = 3). ‡Insufficient exposure to study medication during double-blind phase (*n* = 3); insufficient treatment compliance (*n* = 2); insufficient essential efficacy data (*n* = 1); use of prohibited/prior concomitant medication (*n* = 1). §Withdrew consent (*n* = 3), adverse events (*n* = 2), lost to follow-up (*n* = 1), and administrative reasons (*n* = 1).

Patient disposition is shown in Figure 2. All 58 subjects randomised completed the double-blind phase and entered the OLE; 51 completed the OLE. Demographic characteristics are shown in Table 1. The proportion of patients with total pancreatic resection was higher in the pancreatin group. The majority of patients had undergone surgery for chronic pancreatitis. All patients enrolled were White/Caucasian except for one patient of African heritage in the placebo group.

**Table 1:** Subject characteristics at baseline (safety sample)

	Double-blind phase		OLE
		
	Pancreatin (*n* = 32)	Placebo (*n* = 26)	Pancreatin (*n* = 58)
Age (years), mean ± s.d.	57.6 ± 10.2	59.3 ± 8.7	58.4 ± 9.6
Men, *n* (%)	18 (56.3)	17 (65.4)	35 (60.3)
Weight (kg), mean ± s.d.	68.5 ± 18.1	67.9 ± 12.7	68.0 ± 15.5
BMI (kg/m^2^), mean ± s.d.	23.8 ± 5.5	23.4 ± 4.9	23.5 ± 5.1
Type of pancreatic resection, *n* (%)			
Total	4 (12.5)	0	4 (6.9)
Partial	28 (87.5)	26 (100.0)	54 (93.1)
Whipple/PPPD/equivalent	16 (50.0)	13 (50.0)	29 (50.0)
Duodenal-preserving PHR	7 (21.9)	6 (23.1)	13 (22.4)
Other	5 (15.6)	7 (26.9)	12 (20.7)
Time since surgery (months), mean ±s.d.	57.3 ± 37.9	48.9 ± 41.4	53.4 ± 39.4
Reason for surgery, *n* (%)			
Malignancy	9 (28.1)	5 (19.2)	14 (24.1)
Chronic pancreatitis	23 (71.9)	21 (80.8)	44 (75.9)

PHR, pancreatic head resection; PPPD, pylorus-preserving pancreaticoduodenectomy; OLE, open-label extension; s.d., standard deviation.

The most frequent medical history findings (>20% patients in either group) other than pancreatic surgery/chronic pancreatitis/PEI were diabetes mellitus (28.1% in the pancreatin group and 34.6% in the placebo group), appendectomy (6.3% and 23.1%, respectively) and hypertension (34.4% and 50.0%, respectively). Concomitant medications were used by 88% of subjects in the pancreatin group and 92% of subjects in the placebo group in the double-blind period, the most frequent being drugs for peptic ulcer and gastro-oesophageal reflux disease (38% and 50%, respectively), insulins and analogues (41% and 39%, respectively), and beta-blockers (22% and 23% respectively).

### Efficacy

From baseline to the end of the double-blind period, the CFA increased in the pancreatin group and decreased in the placebo group, resulting in a statistically significant treatment difference (Table 2). These results were confirmed by analysis of the per-protocol sample: treatment difference 32.6% (95% CI: 19.9, 45.4; *P* < 0.001). Statistically significant treatment differences favouring pancreatin were also seen for the changes from baseline in CNA and stool fat (Table 2).

**Table 2:** Change from baseline to end of double-blind phase (full analysis sample)

	Least squares mean (95% CI)			
		
	Pancreatin (*n* = 31)*	Placebo *n* = 25)*	Treatment difference	*P*-value (ancova)
CFA,%	21.4 (13.7, 29.2)	−4.2 (−12.8, 4.5)	25.6 (13.9, 37.3)	<0.001
CNA,%	18.9 (10.6, 27.3)	−10.3 (−19.6, −1.0)	29.2 (16.7, 41.8)	<0.001
Stool fat, g/day	−24.0 (−30.7, −17.3)	6.1 (−1.3, 13.6)	30.2 (40.2, 20.1)	<0.001

ancova, analysis of covariance; CFA, coefficient of fat absorption; CI, confidence interval; CNA, coefficient of nitrogen absorption.

* There were no stool analysis data at the end of the double-blind period for one subject in the pancreatin group and one subject in the placebo group.

Unadjusted values for CFA, CNA and stool characteristics at baseline and end of the double-blind phase are shown in Table 3. Baseline CFA and CNA values were higher in the pancreatin group. Four subjects in the full analysis sample (all randomised to placebo) had negative CFA values, suggesting that fat excretion was greater than fat intake. Their recorded fat excretion and fat intake values were queried but confirmed. Therefore, a sensitivity analysis was also carried out excluding these four patients, which confirmed no change in CFA from baseline to end of double-blind period in the placebo group (Table 3). Additional exploratory analysis indicated that in patients receiving pancreatin, the improvement in CFA was lower in subjects who had undergone surgery due to malignancy compared with those having surgery for chronic pancreatitis (Table 3). Fat and nitrogen intake were similar in both treatment groups at baseline and end of the double-blind phase, with no substantial changes in these parameters in either group (Table 3). Stool frequency decreased by 0.9 stools/day in the pancreatin group and increased by 0.5 stools/day in the placebo group (Table 3).

**Table 3:** CFA, CNA and stool characteristics at baseline and end of double-blind period, unadjusted mean ± s.d. (full analysis sample)

	Pancreatin		Placebo	
		
	Baseline (*n* = 32)	End of double-blind phase (*n* = 31)*	Baseline (*n* = 26)	End of double-blind phase (*n* = 25)*
CFA, %				
Full analysis sample	56.9 ± 17.6	76.6 ± 17.2	49.5 ± 23.5	46.3 ± 31.1
Subjects with positive CFA values	56.9 ± 17.6	76.6 ± 17.2	55.4 ± 19.1§	55.5 ± 18.3¶
By malignancy status				
Malignancy†	54.8 ± 18.9	69.4 ± 23.7	62.7 ± 12.4	46.3 ± 32.4
No malignancy (chronic pancreatitis) ‡	57.8 ± 17.5	79.5 ± 13.3	46.4 ± 24.6	46.3 ± 31.7
CNA, %	55.3 ± 22.2	73.0 ± 16.6	49.6 ± 26.9	39.7 ± 39.0
Stool fat, g/day	44.5 ± 22.4	21.6 ± 12.4	49.3 ± 30.1	55.8 ± 37.3
Stool nitrogen, g/day	6.7 ± 3.5	3.8 ± 2.1	6.7 ± 3.6	8.4 ± 5.3
Fat intake, g/day	104.3 ± 31.5	98.9 ± 31.3**	97.4 ± 33.4	102.5 ± 25.7††
Nitrogen intake, g/day	15.7 ± 7.0	15.0 ± 5.9**	13.6 ± 3.3	14.3 ± 3.2††
Stool weight, g/day	451 ± 210	282 ± 145	487 ± 257	514 ± 268
Number of stools per day	2.5 ± 1.5	1.6 ± 1.2**	2.3 ± 1.7	2.8 ± 1.5

CFA, coefficient of fat absorption; CNA, coefficient of nitrogen absorption; s.d., standard deviation.

* There were no stool analysis data at the end of the double-blind period for one subject in the pancreatin group and one subject in the placebo group.

† Number of patients was nine in pancreatin group and five in the placebo group.

‡ Number of patients was 23 in the pancreatin group and 21 in the placebo group.

§ *n* = 22;

¶ *n* = 21;

** *n* = 32;

†† *n* = 26;

**Figure 3:**
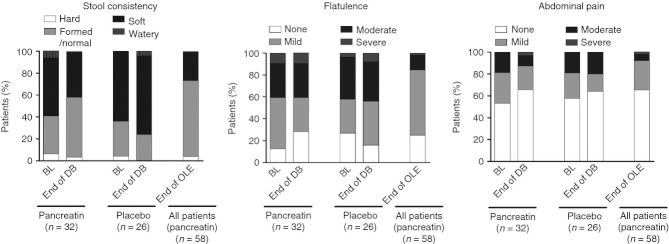
Clinical symptoms. BL, baseline; DB, double-blind; OLE, open-label extension.

Modest improvements in clinical symptoms were observed from baseline to the end of the double-blind period in patients receiving pancreatin (Figure 3). The Clinical Global Impression of disease symptoms, the quality of life component and summary scores, and the health transition score remained stable in both treatment groups during the double-blind period.

**Figure 4:**
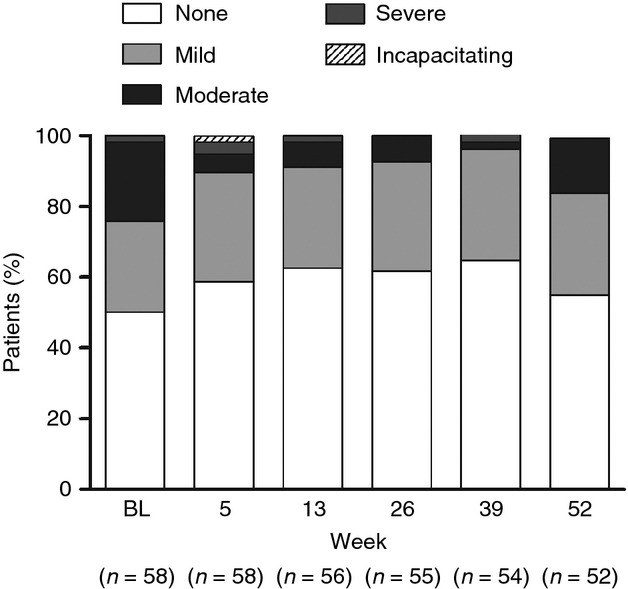
Clinical global impression of disease symptoms. BL, baseline**.**

After 1 year of open-label pancreatin treatment, the mean CFA and CNA values were similar to those seen in the pancreatin group at the end of the double-blind phase. There were statistically significant improvements from baseline to the end of the OLE in CFA, CNA, stool fat/nitrogen and stool weight (Table 4). Statistically significant improvements from baseline were also seen in stool frequency, body weight and BMI (Table 4). Further modest improvements in clinical symptoms were observed at the end of the OLE (Figure 3), with the proportion of subjects with formed/normal stools increasing over time and the proportion of those with moderate/severe flatulence or abdominal pain decreasing over time. There were no relevant changes in laboratory nutritional parameters from baseline to end of OLE. For the Clinical Global Impression of disease symptoms, the percentage of subjects with moderate, severe or incapacitating symptoms decreased until Week 39 but increased again at the end of long-term treatment (Figure 4). At the end of the OLE, slight increases were seen in five of the eight quality of life component scores (bodily pain, general health, vitality, role-emotional, mental health) and in the mental component summary compared with baseline, but none reached statistical significance. The physical functioning component score showed a slight decrease from baseline. The health transition score mean change was −0.40 (*P* = 0.008) at the end of the OLE.

**Table 4:** CFA, CNA, stool characteristics, body weight, and body mass index at baseline and end of the OLE, unadjusted mean ± s.d. (OLE full analysis sample)

	Baseline (*n* = 58)	End (*n* = 48)	*P*-value*
CFA, %	53.6 ± 20.6	78.4 ± 20.7	<0.001
CNA, %	52.8 ± 24.4	74.6 ± 14.0	<0.001
Stool fat, g/day	46.7 ± 26.0	19.1 ± 13.6	<0.001
Stool nitrogen, g/day	6.7 ± 3.5	3.2 ± 1.8	<0.001
Fat intake, g/day†	101.2 ± 32.3	94.4 ± 30.9	n.d.
Nitrogen intake, g/day†	14.8 ± 5.7	13.6 ± 5.8	n.d.
Stool weight, g/day	467 ± 231	235 ± 121	<0.001
Number of stools per day	2.4 ± 1.6	1.5 ± 0.8‡	<0.001
Body weight, kg	68.2 ± 15.8	70.5 ± 16.3§	<0.05
Body mass index, kg/m^2^	23.6 ± 5.2	24.5 ± 5.4§	<0.05

CFA, coefficient of fat absorption; CNA, coefficient of nitrogen absorption; n.d., not determined; OLE, open-label extension; s.d., standard deviation.

* *P*-value is for study end vs. baseline.

† *n* = 50 at end of OLE.

‡ *n* = 52 at end of OLE.

§ *n* = 51 at end of OLE.

**Figure 5:**
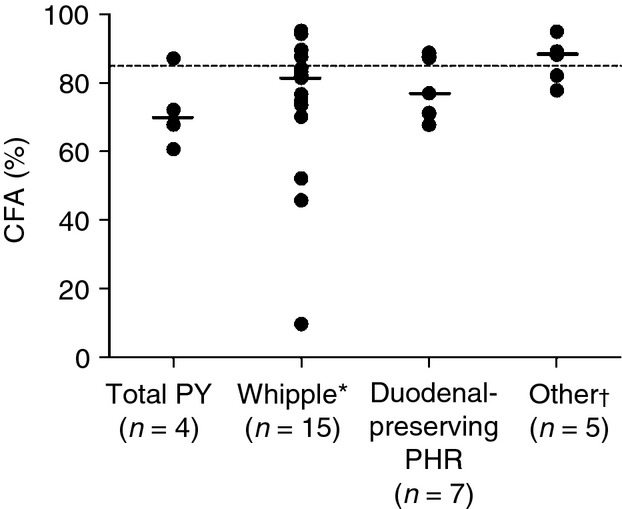
CFA values at the end of double-blind treatment according to type of surgery in patients receiving pancreatin during the double-blind period (*n* = 31; CFA data missing at end of double-blind phase in one patient). Dashed line indicates desired CFA threshold of 85%. Solid line indicates median value in each category. *Whipple procedure or equivalent, e.g. pylorus-preserving pancreaticoduodenectomy. †Pancreatic resection with splenectomy (*n* = 1), distal resection (*n* = 1), partial pancreatectomy due to arterial aneurysm resection (*n* = 1), longitudinal resection (*n* = 1), pancreatic head resection with cholecystectomy (*n* = 1). CFA, coefficient of fat absorption; PHR, pancreatic head resection; PY, pancreatectomy.

A *post-hoc* exploratory analysis of CFA values according to type of surgical procedure was carried out for patients receiving pancreatin in the double-blind phase (Figure 5). Of the patients who had procedures likely to affect intestinal motility and the usual site of exogenous enzyme release (e.g. total pancreatectomy, Whipple procedure or equivalent, duodenal-preserving pancreatic head resection), only 7 of 26 (27%) had on-treatment CFAs >85%. Of those having surgery less likely to affect motility or site of action (e.g. left resection with splenectomy, distal resection, partial resection resulting from arterial aneurysm resection, longitudinal resection, pancreatic head resection with cholecystectomy), the on-treatment CFA was >85% in three of five cases.

### Safety

The mean duration of treatment was 6.6 days pancreatin and 6.7 days placebo in the double-blind phase (range 4–8 days in both groups). The median duration of total exposure to pancreatin was 347 days (range: 44–442).

Adverse events are summarised in Table 5. In the double-blind phase, the overall incidence of adverse events was somewhat higher in the pancreatin group (37.5%) than in the placebo group (26.9%) and the most common was flatulence, surprisingly. There were no adverse events leading to study discontinuation, no serious adverse events and no deaths in the double-blind phase.

**Table 5:** Summary of treatment-emergent adverse events (safety sample)

	Double-blind phase, *n* (%)		Any time*, *n* (%)
	
Pancreatin (*n* = 32)	Placebo (*n* = 26)	(*n* = 58)	
At least one TEAE	12 (37.5)	7 (26.9)	44 (75.9)
At least one TESAE	0	0	15 (25.9)
At least one severe TEAE	2 (6.3)	1 (3.8)	11 (19.0)
TEAEs possibly/probably related to treatment	4 (12.5)	3 (11.5)	8 (13.8)
TEAEs leading to discontinuation	0	0	2 (3.4)
TEAEs occurring in ≥5% patients in any group by preferred term			
Abdominal pain	1 (3.1)	0	9 (15.5)
Diarrhoea	1 (3.1)	1 (3.8)	4 (6.9)
Flatulence	4 (12.5)	2 (7.7)	7 (12.1)
Gamma-glutamyltransferase increased	0	0	4 (6.9)
Headache	2 (6.3)	0	3 (5.2)
Hypertension	1 (3.1)	0	3 (5.2)
Nasopharyngitis	0	1 (3.8)	5 (8.6)
Pyrexia	2 (6.3)	0	7 (12.1)
Vomiting	1 (3.1)	0	3 (5.2)

TEAE, treatment-emergent adverse event; TESAE, treatment-emergent serious adverse event.

* During pancreatin treatment.

Adverse events considered by the investigator as possibly/probably treatment-related occurred in eight (13.8%) subjects at any time during pancreatin treatment (flatulence, diarrhoea, abdominal pain, gastritis, steatorrhoea, headache, renal pain and pruritic rash). Two subjects discontinued during the OLE due to adverse events: one due to diarrhoea (possible relationship to study treatment) and one due to metastases to the peritoneum, which resulted in death (unrelated to study treatment). In the OLE, 15 patients (26%) experienced 27 serious adverse events, but all were considered unrelated or unlikely related to study drug by the Investigator.

No relevant changes were observed overall in laboratory parameters and vital signs, and there were no safety concerns with respect to laboratory parameters and vital signs.

## Discussion

In this population of patients with PEI due to pancreatic surgery, there were statistically significantly greater improvements from baseline to the end of the double-blind phase in fat and nitrogen absorption with pancreatin compared with placebo, as measured by change in CFA and CNA. These improvements translated into only modest improvements in clinical symptoms in the double-blind period, and the Clinical Global Impression of disease symptoms, quality of life and health transition scores remained stable. At the end of the OLE, CFA and CNA values were similar to those observed on pancreatin at the end of the double-blind phase and were significantly improved vs. baseline. Although the investigated dose was effective, it was not sufficient to normalise fat absorption in this study. There was a significant reduction in daily stool frequency and stool weight, and a significant increase in body weight and BMI from baseline to end of the OLE. Modest improvements in clinical symptoms were observed at the end of the OLE, whereas changes in quality of life were variable, with small increases observed in most component scores. There were no relevant changes in laboratory nutritional parameters. The lack of substantial improvement in clinical symptoms and quality of life scores may be related to disease progression (chronic pancreatitis or malignancy), although this cannot be verified as information on progression of disease during the study was not recorded. Alternatively, the lack of improvement may be a consequence of the relatively mild symptoms experienced at baseline in these patients who had been receiving pancreatic enzyme replacement therapy prior to study start. However, improvements in symptoms and quality of life in the OLE phase are probably biased due to the participants' knowledge that they were receiving active treatment, particularly as they were receiving pancreatic enzymes before study entry.

The incidence of adverse events was higher in the pancreatin group than in the placebo group in the double-blind period, with flatulence the most frequent in both groups. Pancreatin was well tolerated; there were no serious adverse events, or adverse events leading to discontinuation, during the randomised period. In the OLE period, there were only two discontinuations due to adverse events (diarrhoea with possible relationship to treatment, and metastases to the peritoneum that was unrelated to treatment). None of the serious adverse events was considered related to study drug. The overall number of adverse events judged to be possibly or probably related to pancreatin treatment was low, and comparable to another long-term study of pancreatin.^14^

The pancreatin dose selected for this study (75 000 Ph. Eur. lipase units per main meal and 50 000 per snack) is an appropriate but rather high starting dose for non-cystic fibrosis patients with PEI, and is within the upper ranges suggested in treatment guidelines/recommendations.^19^–^22^ In a previous study, a pancreatin dose of 72 000 USP lipase units per meal and 36 000 per snack was shown to be effective and well tolerated in patients with PEI due to chronic pancreatitis and pancreatic surgery.^13^ It should be noted that in these clinical studies, patients enrolled had substantial fat malabsorption at baseline (CFA <80%)^23^ and received a high-fat diet during stool collection periods; therefore, higher doses are appropriate. In clinical practice, doses should be individualised based on diet, PEI severity and severity of symptoms. A survey of 91 patients taking pancreatic enzyme replacement therapy in everyday practice to treat PEI following pancreatic surgery for chronic pancreatitis or pancreatic cancer suggested that many patients were under-treated, with a median dose of 6 capsules/day (25 000 lipase units/capsule).^24^ Indeed, the optimal dose of pancreatin should be between the doses used in the above-mentioned studies.

Three previous randomised, double-blind, placebo-controlled trials assessing other pancreatin formulations in patients with PEI due to chronic pancreatitis also showed significant improvements in fat and nitrogen absorption vs. placebo.^13^, ^15^, ^25^ Although the CFA was improved with pancreatin treatment in the present study, the desired CFA threshold of ≥85% was not achieved, and the on-treatment mean CFA value was lower compared with those seen in previous studies (which were 85.5%, 86.1% and 86.6%),^13^, ^15^, ^25^ one of which used a lower pancreatin dose.^26^ Only one of these previous studies enrolled patients with PEI due to pancreatic surgery, but that patient subgroup comprised only 26% of all patients enrolled. Exploratory analysis in that study suggested no difference in pancreatin efficacy in patients having undergone surgery vs. those with chronic pancreatitis.^26^

The lower CFA in the present study is likely due to the differing patient population. In addition to causing PEI, surgical resection may affect intestinal anatomy, physiology, transit time and gastric motility, which could adversely impact pancreatin efficacy. The duodenum is the usual site of exogenous enzyme release and activity, and the primary region for fat digestion and absorption. This is disturbed by major resection involving removal of the duodenum with or without removal of the distal stomach (e.g. pylorus-preserving pancreaticoduodenectomy, Whipple procedure), leading to more distal enzyme release and uneven mixing of pancreatic enzymes and chyme. More than half of all patients in this study had major resections of this type, including four total pancreatectomies in the pancreatin group (compared with zero in the placebo group). These deleterious effects may be of a lesser magnitude in patients undergoing duodenal preserving surgery (e.g. distal pancreatectomy and duodenal-preserving pancreatic head resection). Exploratory analysis according to surgical procedure indicated that the number of patients with an on-treatment CFA ≤85% was higher in those undergoing total or major resection with duodenum removal compared with those undergoing less extensive surgery, although this latter subgroup was small (*n* = 5) (Figure 5). It is possible that patients undergoing major pancreatic resection require higher pancreatic enzyme doses to achieve normalisation of fat digestion because of the anatomical and physiological changes. Further studies are required to determine whether higher doses are more effective in this patient population. Based on the known safety and tolerability profile of pancreatin, higher doses would not be expected to lead to an increase in adverse events. In addition to pancreatic enzyme replacement therapy, dietary counselling and management should be utilised to ensure the intake of a balanced diet, compliance with prescribed dose and correct use of enzymes according to fat intake.^21^ A dietician who is experienced in the management of patients with PEI should be involved.^22^ Alcohol abstinence, frequent small meals, and avoidance of difficult-to-digest foods such as legumes are also recommended.^9^, ^22^ Use of a dietary management strategy in conjunction with a tailored pancreatic enzyme dose may allow normalisation of fat digestion in this patient population.

In the present study, improvements in secondary efficacy outcomes such as CNA, stool frequency, body weight and BMI compare favourably with those of the other pancreatin studies described above.^13^–^15^, ^25^, ^27^ As seen in the long-term OLE of one of the other pancreatin studies,^14^ there were minimal long-term changes in nutritional laboratory parameters in this study. The mean time since surgery was >4 years in the patients enrolled; therefore, this was a relatively stable population. In addition, patients were receiving pancreatic enzyme replacement therapy before the start of the study and therefore were likely to have relatively good nutritional status at baseline, as supported by baseline body weight and BMI values.

The main limitation of this study was that there was no measure of treatment compliance. Furthermore, there was no demarcation of the 72-h stool collection period (i.e. no use of coloured food dye to mark the first and last stools to be collected). Four patients in the placebo group had negative CFA values, suggesting possible errors in nutritional records. However, an analysis excluding patients with negative CFA values supported the results of the primary analysis of no change in CFA values from baseline to end of double-blind treatment in the placebo group. In addition, high levels of undigested fat and secretory diarrhoea may explain negative CFA values in response to secondary inflammatory changes of the intestinal tract.

These findings add to the existing evidence that pancreatin is effective in the treatment of PEI regardless of its aetiology. As the population enrolled had undergone a variety of different pancreatic surgeries due to malignant and non-malignant disease, the results are probably generalisable to all patients undergoing pancreatic resection who require pancreatic enzyme replacement therapy following surgery. Dose titration and monitoring should be considered on an individual basis in the long-term.^28^ In conclusion, this study demonstrates the superior efficacy of pancreatin 25000 minimicrospheres over placebo in patients with PEI after pancreatic surgery. In addition, pancreatin was generally well tolerated at the strength and dosage administered.

## Authorship

*Guarantor of the article*: Christoph M. Seiler, MD, MSc.

*Author contributions*: HF contributed to the design of the study. All authors were involved in data acquisition and interpretation, and drafting or critically reviewing/revising the manuscript. All authors approved the final version of this article.
